# Colossal barocaloric effects in the complex hydride Li$_{2}$B$_{12}$H$_{12}$

**DOI:** 10.1038/s41598-021-91123-4

**Published:** 2021-06-07

**Authors:** Kartik Sau, Tamio Ikeshoji, Shigeyuki Takagi, Shin-ichi Orimo, Daniel Errandonea, Dewei Chu, Claudio Cazorla

**Affiliations:** 1 Mathematics for Advanced Materials-Open Innovation Laboratory (MathAM-OIL), National Institute of Advanced Industrial Science and Technology (AIST), Tohoku University, Sendai, 980-8577 Japan; 2 Institute for Materials Research, Tohoku University, Sendai, 980-8577 Japan; 3 Advanced Institute for Materials Research, Tohoku University, Sendai, 980-8577 Japan; 4 Departament de Física Aplicada, Institut de Ciència de Materials, MALTA Consolider Team, Universitat de València, Edifici d’Investigació, 46100 Burjassot, Spain; 5 School of Materials Science and Engineering, UNSW Sydney, Sydney, NSW 2052 Australia; 6 Departament de Física, Universitat Politècnica de Catalunya, Campus Nord B4-B5, 08034 Barcelona, Spain

**Keywords:** Condensed-matter physics, Materials science, Materials for energy and catalysis

## Abstract

Traditional refrigeration technologies based on compression cycles of greenhouse gases pose serious threats to the environment and cannot be downscaled to electronic device dimensions. Solid-state cooling exploits the thermal response of caloric materials to changes in the applied external fields (i.e., magnetic, electric and/or mechanical stress) and represents a promising alternative to current refrigeration methods. However, most of the caloric materials known to date present relatively small adiabatic temperature changes ($|\Delta T| \sim 1$ to 10 K) and/or limiting irreversibility issues resulting from significant phase-transition hysteresis. Here, we predict by using molecular dynamics simulations the existence of colossal barocaloric effects induced by pressure (isothermal entropy changes of $|\Delta S| \sim 100$ J K$^{-1}$ kg$^{-1}$) in the energy material Li$_{2}$B$_{12}$H$_{12}$. Specifically, we estimate $|\Delta S| = 367$ J K$^{-1}$ kg$^{-1}$ and $|\Delta T| = 43$ K for a small pressure shift of *P* = 0.1 GPa at $T = 480$ K. The disclosed colossal barocaloric effects are originated by a fairly reversible order–disorder phase transformation involving coexistence of Li$^{+}$ diffusion and (BH)$_{12}^{-2}$ reorientational motion at high temperatures.

Solid-state cooling is an environmentally friendly, energy efficient, and highly scalable technology that can solve most of the problems associated with conventional refrigeration methods based on compression cycles of greenhouse gases (i.e., environmental harm and lack of downsize scaling). Upon application of small or moderate magnetic, electric and/or mechanical stress field shifts good caloric materials undergo noticeable temperature changes ($|\Delta T| \sim 1$ to 10 K) as a result of induced phase transformations that involve large entropy variations ($|\Delta S| \sim 100$ to 100 J K$^{-1}$ kg$^{-1}$)^1–11^. For instance, the archetypal elastocaloric compound NiTi exhibits an adiabatic temperature change of $\approx \, 40$ K/GPa^12,13^ and the eminent magnetocaloric crystal Gd$_{5}$Si$_{2}$Ge$_{2}$ a $|\Delta T|$ of $\approx 3$ K/T^14^. Solid-state cooling capitalizes on such substantial caloric effects to engineer refrigeration cycles. From a performance point of view, that is, largest $|\Delta T|$ and $|\Delta S|$ (although these are not the only parameters defining cooling efficiency^15,16^), mechanocaloric effects driven by small uniaxial stresses (i.e., elastocaloric effects) and hydrostatic pressure (i.e., barocaloric effects) appear to be the most promising^1–3,7,9–13^.

**Figure 1:**
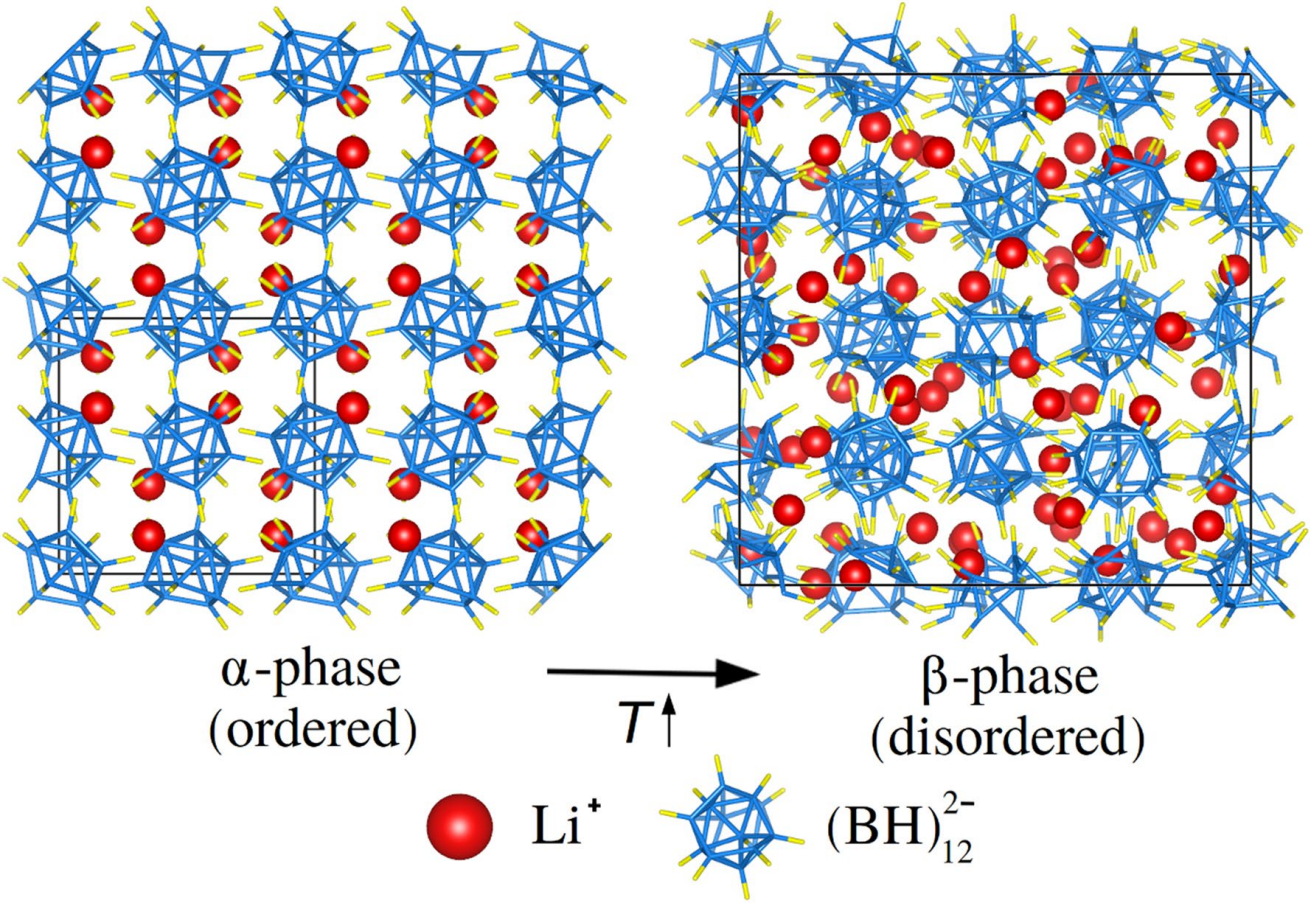
Low-*T* (ordered) and high-*T* (disordered) phases of bulk Li$_{2}$B$_{12}$H$_{12}$. The low-*T* phase ($\alpha$) presents cubic symmetry and space group $Pa\overline{3}$^30^. In the high-*T* phase ($\beta$), cubic symmetry is preserved but the Li$^{+}$ ions are highly mobile and the (BH)$_{12}^{-2}$ icosahedra present reorientational disorder^32^. The *T*-induced $\alpha \rightarrow \beta$ phase transition is an order–disorder isosymmetric transformation that is regarded as of first order^32^. Li, B, and H ions are represented with red, blue, and yellow colours, respectively.

Recently, colossal barocaloric effects (defined here as $|\Delta S| \sim \,100$ J K$^{-1}$ kg$^{-1}$) have been measured in two different families of materials that display intriguing order–disorder phase transitions^17–19^. First, giant barocaloric effects have been theoretically predicted^20^ and experimentally observed in the archetypal superionic compound AgI^17^. AgI exhibits a first-order normal (low-entropy) to superionic (high-entropy) phase transition that responds to both temperature and pressure^21–23^ and which involves the presence of highly mobile silver ions in the high-*T* superionic state^24,25^. Likewise, the entropy changes estimated for other superionic phase transitions are also large in general^26–29^. And second, colossal barocaloric effects have been reported for the molecular solid neopentylglycol^18,19^, (CH$_{3}$)$_{2}$C(CH$_{2}$OH)$_{2}$, and other plastic crystals^15^. In these solids, molecules reorient almost freely around their centers of mass, which remain localized at well-defined lattice positions. Molecular rotations lead to orientational disorder, which renders high entropy. By using hydrostatic pressure, it is possible to block such molecular reorientational motion and thus induce a fully ordered state characterized by low entropy^29^. The barocaloric effects resulting from this class of first-order order–disorder phase transitions are huge and comparable in magnitude to those achieved in conventional refrigerators with environmentally harmful gases^15,18,19^.

Here, we report the prediction of colossal barocaloric effects ($|\Delta S| \sim 100$ J K$^{-1}$ kg$^{-1}$) in the energy material Li$_{2}$B$_{12}$H$_{12}$ (LBH), a complex hydride that is already known from the fields of hydrogen storage^30,31^ and solid-state batteries^32–34^. By using molecular dynamics simulations, we identify a pressure-induced isothermal entropy change of $|\Delta S| = 367$ J K$^{-1}$ kg$^{-1}$ and an adiabatic temperature change of $|\Delta T| = 43$ K at $T = 480$ K. These colossal entropy and temperature changes are driven by small hydrostatic pressure shifts of $\sim \, 0.1$ GPa, thus yielding gigantic barocaloric strengths of $|\Delta S| / P \sim 10^{3}$ J K$^{-1}$ kg$^{-1}$ GPa$^{-1}$ and $|\Delta T| / P \sim 10^{2}$ K GPa$^{-1}$. The colossal barocaloric effects disclosed in bulk LBH are originated by simultaneous frustration of Li$^{+}$ diffusion and (BH)$_{12}^{-2}$ icosahedra reorientational motion driven by hydrostatic pressure. Thus, alkali-metal complex borohydrides ($A_{2}$B$_{12}$H$_{12}$, $A =$ Li, Na, K, Cs^35,36^) emerge as a promising new family of caloric materials in which the salient phase-transition features of fast-ion conductors and plastic crystals coexist and cooperate to render colossal barocaloric effects.

**Figure 2:**
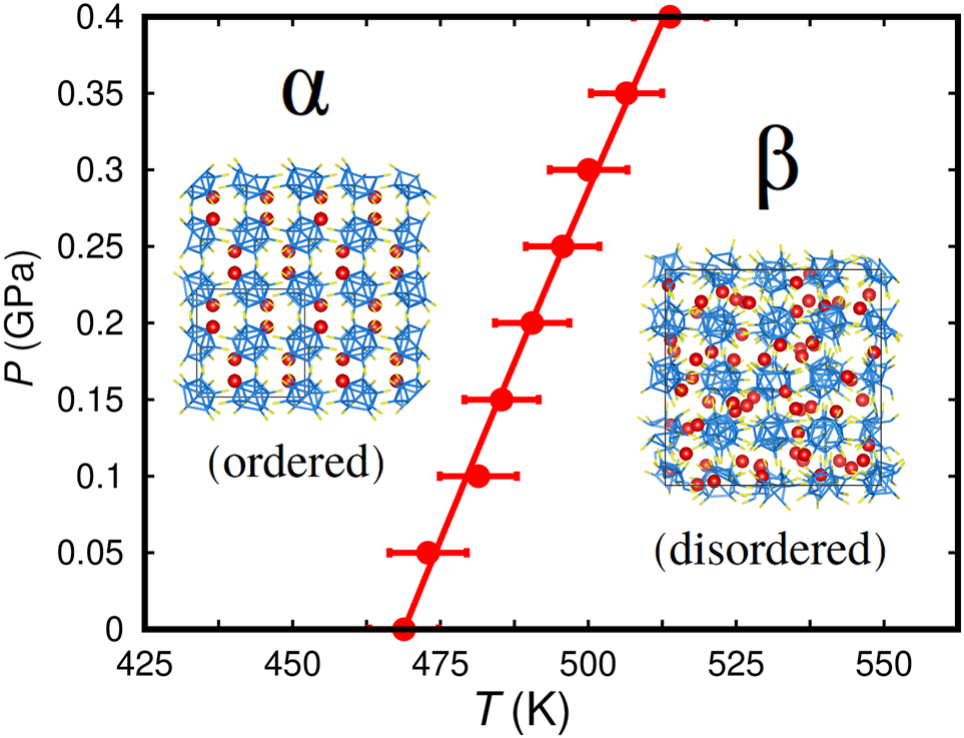
Estimated Li$_{2}$B$_{12}$H$_{12}$ phase diagram expressed as a function of pressure (*P*) and temperature (*T*). Results were obtained from classical molecular dynamics simulations performed with the LBH force field reported in work^38^. The coexistence line separating the stability region of the $\alpha$ and $\beta$ phases, represented with a solid red line, was determined through a polynomial fit to the theoretical phase-transition data (solid red dots and error bars) and corresponds to the analytical expression $T_{\alpha \leftrightarrow \beta } (P) = 468.98 + 103.96 \cdot P + 13.97 \cdot P^{2}$, where the temperature and pressure are respectively expressed in units of K and GPa. Error bars represent statistical uncertainties estimated with time-average techniques (“Methods”).

## Results

At ambient conditions, lithium dodecahydrododecaborate (Li$_{2}$B$_{12}$H$_{12}$), LBH, presents an ordered cubic $Pa\overline{3}$ phase ($Z = 4$), referred to as $\alpha$ hereafter, which is characterized by Li$^{+}$ cations residing on near-trigonal-planar sites surrounded by three (BH)$_{12}^{-2}$ icosahedron anions. In turn, each (BH)$_{12}^{-2}$ anion resides in an octahedral cage surrounded by six Li$^{+}$ cations (Fig. 1)^30^. A symmetry preserving order–disorder phase transition occurs at high temperatures ($\sim \, 600$ K) that stabilises a disordered state, referred to as $\beta$ hereafter, in which the Li$^{+}$ cations are mobile and the (BH)$_{12}^{-2}$ anions present reorientational motion (Fig. 1; Supplementary Fig. 1)^32,38^. The relative volume expansion that has been experimentally measured for such a first-order order–disorder phase transition is $\Delta V^\text{expt} / V_{\alpha }^\text{expt} \approx \,8$%^32^ and the corresponding phase transition enthalpy $\Delta H^\text{expt} \approx 130$ kJ kg$^{-1}$^37^. The huge volume and enthalpy variations accompanying the $\alpha \leftrightarrow \beta$ transformation could be promising in the context of barocaloric effects if the involved phase transition was responsive to small hydrostatic pressure shifts of $\sim \,0.1$ GPa. To the best of our knowledge, this possibility has never been hitherto explored. We performed classical molecular dynamics (MD) simulations based on a recently proposed LBH force field^38^ to fill up such a knowledge gap (“Methods” and Supplementary Methods) and thus assess the potential of complex hydrides as barocaloric materials.

**Figure 3:**
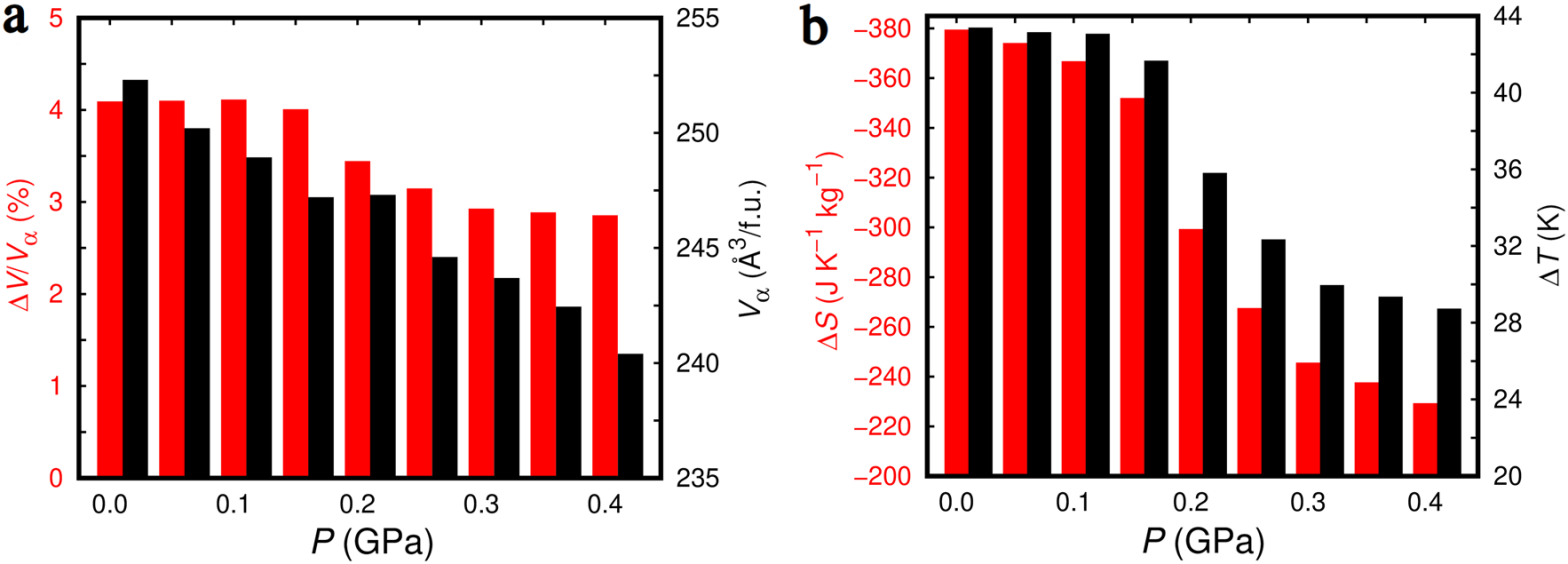
Effect of pressure on the structural and barocaloric properties of Li$_{2}$B$_{12}$H$_{12}$ estimated with classical molecular dynamics techniques and the force field reported in work^38^. (**a**) Relative volume change associated with the *T*-induced $\alpha \rightarrow \beta$ phase transition expressed as a function of pressure (red); volume of the $\alpha$ phase per formula unit (f.u.), $V_{\alpha }$, at the phase-transition conditions (black). (**b**) Isothermal entropy (red) and adiabatic temperature (black) changes associated with the barocaloric response of bulk Li$_{2}$B$_{12}$H$_{12}$ expressed as a function of pressure.

Figure 2 shows the *P*–*T* phase diagram of bulk LBH theoretically estimated with MD techniques (“Methods”). We determined the coexistence line of the $\alpha$ and $\beta$ phases by conducting numerous MD simulations at small *P*–*T* shifts of 0.05 GPa and 12.5 K, and by monitoring the structural, Li$^{+}$ diffusion, and (BH)$_{12}^{-2}$ reorientational properties of the system (“Methods”). In consistency with the experiments, a point in the $\alpha$–$\beta$ coexistence line of Fig. 2 was identified by sharp and simultaneous changes in the volume, Li$^{+}$ diffusion coefficient ($D_\text{Li}$), and (BH)$_{12}^{2-}$ reorientational frequency ($\lambda _{\text{B}_{12}\text{H}_{12}}$) of bulk LBH (errorbars in Fig. 2 correspond to statistical uncertainties obtained through time averages). It was found that the critical temperature of the $\alpha \leftrightarrow \beta$ transformation can be certainly modified by hydrostatic pressure. Specifically, $T_{\alpha \leftrightarrow \beta }$ increases under increasing pressure at an approximate rate of $\sim 100$ K GPa$^{-1}$; a simple second-order polynomial fit to our set of predicted $\alpha$–$\beta$ coexistence points renders the analytical expression $T_{\alpha \leftrightarrow \beta } (P) = 468.98 + 103.96 \cdot P + 13.97 \cdot P^{2}$, in which the temperature and pressure are respectively expressed in units of K and GPa.

The predicted volume change associated with the $\alpha \leftrightarrow \beta$ phase transition expressed as a function of pressure are shown in Fig. 3a. At zero pressure, our MD simulations render a transition volume change, $\Delta V$, relative to the volume of the $\alpha$ phase at $T_{\alpha \leftrightarrow \beta }$ (namely, $V_{\alpha } = 252.3$ Å$^{3}$ per formula unit—f.u.) of $\approx +4$%. This result significantly underestimates the corresponding experimental value of $\approx +8$%^32^. The main reason for this discrepancy is that the employed force field is not perfect and overestimates the zero-pressure volume of the $\alpha$ and $\beta$ phases at $T_{\alpha \leftrightarrow \beta }$ by $\approx 10$% and 5% (e.g., $V_{\alpha }^\text{expt} = 231.0$ Å$^{3}$/f.u.^32^), respectively. Also, it should be noted that in our simulations we considered perfect and stoichiometric bulk LBH configurations whereas in real samples crystal defects like vacancies, grain boundaries and dislocations are always present; such crystal defects typically have little effect on standard first-order phase transitions (i.e., the agreement between experimental results obtained for single crystals and polycrystalline samples is consistent most of the times) but they are known to play a critical role on the physical properties of fast-ion conductors like LBH^39–41^.

Nevertheless, in spite of the $\Delta V / V_{\alpha }$ differences between our MD results and experiments, which to some extent were expected and are insurmontable, our theoretical estimations for bulk LBH can be regarded overall as physically meaningful and reasonably accurate. For example, by using simple thermodynamic relations, the $T_{\alpha \leftrightarrow \beta }$ analytical expression reported above and the $\Delta V$ results enclosed in Fig. 3a, we estimated a zero-pressure transition enthalpy of $\Delta H \approx 170$ kJ kg$^{-1}$, which is fairly consistent with the reported experimental value of $\Delta H^\text{expt} \approx 130$ kJ kg$^{-1}$^37^. Moreover, as it is shown in work^38^ and the Supplementary Discussion, the employed interatomic potential satisfactorily reproduces the general structural and dynamical properties (i.e., Li$^{+}$ ion diffusion and (BH)$_{12}^{-2}$ icosahedra reorientational motion) of bulk LBH as determined from experiments and first-principles molecular dynamics simulations based on density functional theory (which are *virtually* free of the limitations affecting interatomic potentials^42,43^).

Therefore, we proceeded to estimate the barocaloric isothermal entropy, $\Delta S$, and adiabatic temperature, $\Delta T$, changes induced by pressure shifts of $0 \le P \le 0.4$ GPa in bulk LBH. For this end, and in view of the first-order nature of the $\alpha \leftrightarrow \beta$ phase transformation^32^, we employed the Clausius–Clapeyron method^2,3^, which involves the knowledge of the $\alpha$–$\beta$ coexistence line slope (Fig. 2), and the $\Delta V$ (Fig. 3a) and heat capacity (Supplementary Fig. 2) data estimated for bulk LBH as a function of pressure and temperature (“Methods”). The resulting $\Delta S$ and $\Delta T$ values enclosed in Fig. 3b in fact render colossal barocaloric effects. For example, at $T = 480$ K and $P = 0.1$ GPa we calculated an isothermal entropy change of $-367$ J K$^{-1}$ kg$^{-1}$ and an adiabatic temperature change of $+43$ K. The size of $\Delta S$ and $\Delta T$ were found to gradually decrease under pressure (e.g., at $T = 515$ K and $P = 0.4$ GPa we estimated $-229$ J K$^{-1}$ kg$^{-1}$ and $+28$ K, respectively). Meanwhile, the predicted LBH barocaloric effects are conventional, that is, $\Delta T > 0$, because the low-entropy ordered phase ($\alpha$) is stabilized over the high-entropy disordered phase ($\beta$) as a result of applying pressure ($\Delta S < 0$). In “Discussion” section, we will compare the barocaloric performance of LBH with those of other well-known barocaloric materials. In what follows, the atomistic origins of the extraordinary $\Delta S$ and $\Delta T$ values reported in Fig. 3b are discussed.

**Figure 4:**
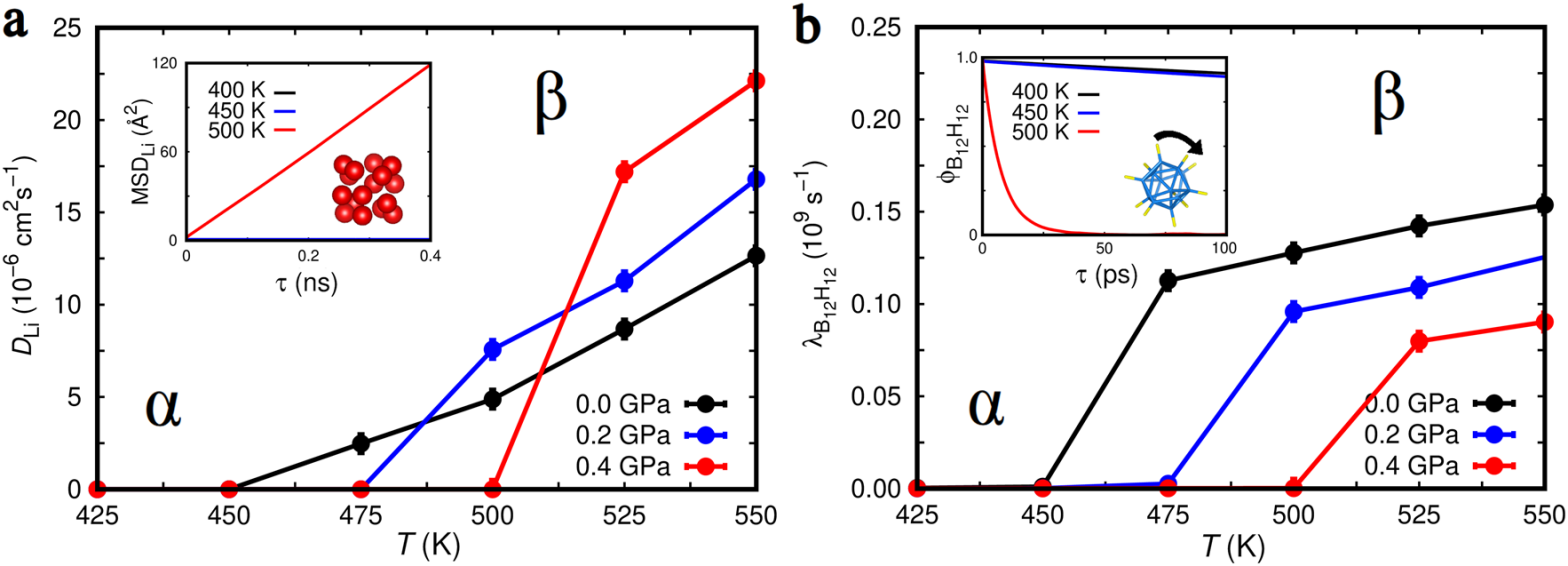
Order parameter changes associated with the *T*-induced $\alpha \rightarrow \beta$ phase transition occurring in bulk Li$_{2}$B$_{12}$H$_{12}$ at different pressures (estimated with classical molecular dynamics techniques and the force field reported in work^38^). (**a**) Computed lithium ion diffusion coefficient, $D_\text{Li}$, expressed as a function of temperature and pressure. The inset shows the Li mean-squared displacement (MSD$_\text{Li}$) data employed for the calculation of $D_\text{Li}$ at $P = 0.2$ GPa (“Methods”). (**b**) Estimated (BH)$_{12}^{-2}$ icosahedra reorientational rate, $\lambda _{\text{B}_{12}\text{H}_{12}}$, expressed as a function of temperature and pressure. The inset shows the (BH)$_{12}^{-2}$ icosahedra angular auto-correlation function ($\phi _{\text{B}_{12}\text{H}_{12}}$) data employed for the calculation of $\lambda _{\text{B}_{12}\text{H}_{12}}$ at $P = 0.2$ GPa (“Methods”).

There are two possible sources of large entropy variation in LBH, one stemming from Li$^{+}$ ionic diffusion and the other from (BH)$_{12}^{-2}$ icosahedra reorientational motion. When hydrostatic pressure is applied on the disordered $\beta$ phase and the ordered $\alpha$ phase is stabilized, both ionic diffusion and molecular orientational disorder are drastically reduced, thus the entropy of the crystal significantly decreases. This conclusion is straightforwardly deduced from the *P*-induced variation of the Li$^{+}$ diffusion coefficient, $D_\text{Li}$, and reorientational (BH)$_{12}^{-2}$ frequency, $\lambda _{\text{B}_{12}\text{H}_{12}}$, shown in Fig. 4a,b (“Methods”). For instance, at $T = 475$ K and zero pressure $D_\text{Li}$ and $\lambda _{\text{B}_{12}\text{H}_{12}}$ amount to $2.5 \times 10^{-6}$ cm$^{2}$s$^{-1}$ and $1.2 \times 10^{8}$ s$^{-1}$, respectively, whereas at $P = 0.2$ GPa and same temperature both quantities are practically zero. (It is noted in passing that the theoretical $\lambda _{\text{B}_{12}\text{H}_{12}}$ value computed at zero pressure and $T \approx T_{\alpha \leftrightarrow \beta }$ is in very good agreement with the corresponding experimental value of $1.5 \times 10^{8}$ s$^{-1}$^37^.) The two resulting entropy change contributions are large and of the same sign, thus they sum up giving rise to colossal $|\Delta S|$ values (Fig. 3b).

Which of these two *P*-induced order-restoring effects is most relevant for the barocaloric performance of bulk LBH? To qualitatively answer this question, we performed constrained MD simulations in which we forced the Li$^{+}$ and (BH)$_{12}^{-2}$ ions to remain localized around their equilibrium positions at any temperature during separate MD runs (Supplementary Fig. 3). This type of artificial conditions in principle cannot be imposed in the experiments but can be easily enforced in the atomistic simulations (e.g., by disproportionately increasing the mass of the targeted ions). Specifically, we assumed that the phase transition entropy change could be decomposed in the sum of terms: $\Delta S = \Delta S_\text{Li} + \Delta S_{\text{B}_{12}\text{H}_{12}} + \Delta S_\text{Li} \vert B_{12}H_{12}$, where the first term corresponds to *pure* Li$^{+}$ contributions (obtained by *freezing* the (BH)$_{12}^{-2}$ anions), the second to *pure* (BH)$_{12}^{-2}$ contributions (obtained by *freezing* the Li$^{+}$ cations), and the third to correlated Li$^{+} \vert$(BH)$_{12}^{-2}$ contributions (obtained by substracting the sum of the *pure* terms $\Delta S_\text{Li}$ and $\Delta S_{\text{B}_{12}\text{H}_{12}}$ to $\Delta S$). The partial entropy change values were obtained by considering the Clausius-Clapeyron relation, the volume changes obtained from constrained MD simulations, and the $\alpha$–$\beta$ coexistence line obtained from standard MD simulations. The entropy contributions estimated by following this approximate $\Delta S$ partition ansatz are shown in Supplementary Fig. 3. It was found that mixed ionic-molecular Li$^{+} \vert$(BH)$_{12}^{-2}$ correlations contribute the most to the $\alpha \leftrightarrow \beta$ phase transition entropy change. In particular, *pure* Li$^{+}$ entropy change contributions amount to $\approx 10$%, *pure* (BH)$_{12}^{-2}$ to $\approx 1$ to 5% (depending on pressure), and mixed Li$^{+} \vert$(BH)$_{12}^{-2}$ to $\approx 90$ to 85%. This result is not surprising since the interplay between ionic diffusion and molecular reorientational motion in LBH^38^ and other akin complex materials^44^ typically is very strong.

Figure 4a shows that at $T \gtrsim 525$ K the diffusivity of Li$^{+}$ ions increases under increasing pressure. For example, at $T = 525$ K and zero pressure we estimate $D_\text{Li} = 8.7 \times 10^{-6}$  cm$^{2}$ s$^{-1}$ whereas at $P = 0.4$ GPa and same temperature we obtain $17.2 \times 10^{-6}$ cm$^{2}$ s$^{-1}$. This ionic diffusion behaviour is highly anomalous since hydrostatic compression typically hinders ionic transport^23,25,26^. On the other hand, the reorientational motion of the (BH)$_{12}^{-2}$ icosahedra behaves quite normally, that is, decreases under pressure^3,15^. For instance, at $T = 525$ K and zero pressure we estimate $\lambda _{\text{B}_{12}\text{H}_{12}} = 1.4 \times 10^{8}$ s$^{-1}$ whereas at $P = 0.4$ GPa and same temperature we obtain $0.7 \times 10^{8}$ s$^{-1}$ (Fig. 4b). In view of the physical insight previously gathered on $\Delta S$, we hypothesized that such anomalous *P*-induced lithium diffusion enhancement could be related to the strong interplay between Li$^{+}$ and (BH)$_{12}^{-2}$ ions. In particular, high anionic reorientational motion could make the (BH)$_{12}^{-2}$ centers of mass (CMs) to fluctuate wildly thus blocking partially the ionic current channels^44^. Consistent to this hypothesis, and for small and moderate pressures, large (BH)$_{12}^{-2}$ CMs fluctuations should be accompanied by reduced Li$^{+}$ diffusivity while small (BH)$_{12}^{-2}$ CMs fluctuations should be accompanied by enhanced Li$^{+}$ diffusivity. The *P*-dependent MD results obtained for the fluctuations of anionic CMs shown in Supplementary Fig. 4 appear to confirm this hypothesis. Specifically, at high temperatures and $P = 0.4$ GPa the fluctuations of the (BH)$_{12}^{-2}$ CMs are about 5% smaller than estimated at zero pressure and same temperature, which may explain the origin of the larger $D_\text{Li}$’s found in the former case. Nevertheless, the identified anomalous lithium diffusivity ceases at $P \approx 0.6$ GPa since under higher pressures $D_\text{Li}$ gradually decreases (Supplementary Fig. 5). Analysis of such anomalous *P*-induced Li$^{+}$ diffusion enhancement effect predicted by MD simulations deserves further theoretical exploration using highly accurate but computationally expensive first-principles methods (which is out of the scope of the present study).

**Table 1:** Materials presenting giant ($|\Delta S| \sim 10$ J K$^{-1}$ kg$^{-1}$) and colossal ($|\Delta S| \sim 100$ J K$^{-1}$ kg$^{-1}$) barocaloric effects.

	$T \text{(K)}$	$P \text{(GPa)}$	$|\Delta S| \text{(J K}^{-1} \text{kg}^{-1})$	$|\Delta T| \text{(K)}$	$|\Delta S|/P \text{(J K}^{-1} \text{kg}^{-1} GPa^{-1})$	$|\Delta T|/P \text{(K~GPa}^{-1})$	$\text{Material}$
$\text{Ni}_{51}\text{Mn}_{33}\text{In}_{16}$	330	0.25	41.0	4.0	164	16.0	$\text{SMA}$^45^ (Expt.)
$\text{Fe}_{49}\text{Rh}_{51}$	310	0.11	12.5	8.1	114	73.6	$\text{SMA}$^46^ (Expt.)
$\text{Mn}_{3}\text{XN}$	262–290	$\sim 0.10$	$\sim 30$	$\sim 5$	$\sim 300$	$\sim 50$	$\text{AMA}$^47,48^ (Expt.)
$\text{(NH}_{4})_{2}\text{SO}_{4}$	220	0.10	130.0	8.0	1300	80.0	$\text{FE}$^49^ (Expt.)
$\text{[TPrA][Mn(dca)}_{3}]$	330	0.01	30.5	4.1	3050	410.0	$\text{OIH}$^50^ (Expt.)
$\text{[FeL}_{2}][\text{BF}_{4}]_{2}$	262	0.03	80.0	3.0	2667	100.0	$\text{MC}$^54^ (Expt.)
$\text{(CH}_{3})_{2}\text{C(CH}_{2}\text{OH})_{2}$	320	0.25	445.0	30–45	1780	120–180	$\text{MC}$^18,19^ (Expt.)
$\text{AgI}$	400	0.25	62.0	36.0	248	144.0	$\text{FIC}$^17^ (Expt.)
$\text{Li}_{2}\text{B}_{12}\text{H}_{12}$	480	0.10	367.0	43.0	3670	430.0	$\text{FIC/MC}~\text{(Theory)}$

*T* working temperature, *P* applied pressure, $|\Delta S|$ isothermal entropy change, $|\Delta T|$ adiabatic temperature change, $|\Delta T|/P$ barocaloric strength, *SMA* shape-memory alloy, *AMA* antiferromagnetic metallic alloy (X = Ga,Ni), *FE* ferroelectric, *OIH* organic–inorganic hybrid perovskite, *MC* molecular crystal, *FIC* fast-ion conductor. All references correspond to experimental works (Expt.) except for the present computational study (Theory).

## Discussion

To date, large barocaloric effects have been experimentally measured for a number of shape-memory alloys^45,46^, antiferromagnetic metallic alloys^47,48^, polar compounds^49^, organic–inorganic hybrid perovskites^50,51^, fluoride-based materials^52^, polymers^53^, the fast-ion conductor AgI^17^ and molecular crystals^18,19,54^. In Table 1, we compare the predicted barocaloric performance of bulk LBH with the experimentally measured barocaloric performance of some representative materials^1–3^. It is noted that the $\Delta S$ and $\Delta T$ values reported here for LBH do not take into account the likely existence of adverse hysteresis and reversibility effects (see below), which tend to reduce significantly their size and have been included in some experimental studies^17,19,45,46,49^. Therefore, comparison between our theoretical predictions and experimental results may be regarded as indicative in some cases.

The isothermal entropy change induced in LBH by a small hydrostatic pressure of 0.1 GPa, 367 J K$^{-1}$ kg$^{-1}$, is comparable in magnitude to the record $|\Delta S|$ that has been recently reported for the plastic crystal neopentylglycol by considering a similar pressure shift, namely, $\approx 500$ J K$^{-1}$ kg$^{-1}$^18,19^. The rest of materials in Table 1 present isothermal entropy changes that are appreciably smaller, made the exception of the polar crystal (NH$_{4}$)$_{2}$SO$_{4}$ which registers 130 J K$^{-1}$ kg$^{-1}$. As regards $|\Delta T|$, the clear contestants of LBH are the fast-ion conductor AgI (36 K)^17^ and again the plastic crystal (CH$_{3}$)$_{2}$C(CH$_{2}$OH)$_{2}$ (30–45 K)^18,19^. In terms of the barocaloric strengths $\text{BSS}$ and $\text{BST}$, defined as $\text{BSS} \equiv |\Delta S| / P$ and $\text{BST} \equiv |\Delta T| / P$, LBH is placed amongst the best performers. For instance, the organic–inorganic hybrid perovskite [TPrA] [Mn(dca)$_{3}$] displays the largest experimental BSS and BST coefficients of all crystals, $\approx 3000$ J K$^{-1}$ kg$^{-1}$ GPa$^{-1}$ and $\approx 400$ K GPa$^{-1}$, which are comparable in magnitude to those predicted for bulk LBH. Likewise, the experimental barocaloric strengths reported for the plastic crystal neopentylglycol are close to those predicted for LBH, which hints at the common order–disorder origin of their relevant phase transformations.

As it was mentioned in the Introduction, the size of the $|\Delta T|$ and $|\Delta S|$ shifts are not the only parameters defining the barocaloric performance of a material. The degree of reversibility of the involved *P*-induced phase transition, for instance, is another important barocaloric descriptor that provides information on the materials efficiency during successive pressure application and removal cycles. Specifically, the hysteresis of the transition makes the materials behaviour to depend on its cycling history and to increase the value of the external field that is required to bring the phase transition to completion^15,16^. As a consequence, the barocaloric performance of a hysteretic material can be significantly worse than that of its ideal non-hysteretic counterpart. In order to quantify the degree of reversibility associated with the $\alpha \leftrightarrow \beta$ phase transition in LBH, we performed a series of long MD simulations ($\sim 4$ ns) in which the pressure was kept fixed while the temperature was varied steadily first from 425 up to 625 K and subsequently from 625 back to 425 K. The results of these simulations indicate that the degree of reversibility of the $\alpha \leftrightarrow \beta$ order–disorder phase transition is fairly good (Supplementary Fig. 6). In particular, by monitoring the variation of the system volume we found that at zero pressure the difference between the transition temperatures observed during the heating and cooling switches was $\Delta T_{h} \equiv T_{\alpha \rightarrow \beta } - T_{\beta \rightarrow \alpha } \approx 50$ K. This predicted temperature hysteresis value is in reasonably good agreement with the corresponding experimental value of $\approx 20$ K^37^. According to our simulations, however, the hysteresis of the $\alpha \leftrightarrow \beta$ transformation tends to increase under increasing pressure (e.g., $\Delta T_{h} \approx 100$ K at $P = 0.4$ GPa). It is worth noting that, due to obvious computational limitations, our $\Delta T_{h}$ estimation should be regarded as tentative since the temperature change rate adopted in our simulations ($\sim 10^{11}$ K/s) is orders of magnitude faster than in the real experiments ($\sim$ K/s), and in practice phase-transition hysteresis may depend strongly on this parameter.

Arguably the only weakness of bulk LBH in terms of barocaloric potential is that the critical temperature of the order–disorder $\alpha \rightarrow \beta$ phase transition is significantly higher than room temperature. However, this practical problem can be solved effectively by means of doping and alloying strategies. In fact, recently it has been experimentally shown that carbon-substituted LBH, LiCB$_{11}$H$_{12}$, presents a much lower $\alpha \rightarrow \beta$ transition temperature of $\approx 400$ K^55^, and that the disordered $\beta$ phase is already stabilized at room temperature in structurally similar Li(CB$_{9}$H$_{10}$)–Li(CB$_{11}$H$_{12}$) solid solutions^56^. Moreover, the type of isosymmetric order–disorder phase transition underlying the exceptional barocaloric behaviour of LBH also occurs in analogous alkali–metal complex hydrides (A$_{2}$B$_{12}$H$_{12}$, A = Na, K, Cs)^57^ and other earth-abundant and non-toxic materials like KHPO$_{4}$, NaAlSi$_{3}$O$_{8}$ and KNO$_{3}$^58,59^. Thus the present theoretical study should motivate experimental searches of colossal barocaloric effects in LBH and related materials exhibiting lower transition temperatures, so that they can be employed in technological solid-state refrigeration applications.

In conclusion, we have predicted the existence of colossal barocaloric effects rendering isothermal entropy changes of $\sim 100$ J K$^{-1}$ kg$^{-1}$ and adiabatic temperature changes of $\sim 10$ K in the complex hydride Li$_{2}$B$_{12}$H$_{12}$, which are driven by moderate pressure shifts of $\sim 0.1$ GPa. The phase transition underlying such colossal barocaloric effects is remarkable as it combines key ingredients of fast-ion conductors (i.e., ionic diffusion) and molecular crystals (i.e., reorientational motion), materials that individually have been proven to be excellent barocaloric materials. This same type of isosymmetric order–disorder phase transition is likely to occur in other economically affordable and innocuous compounds (e.g., Na$_{2}$B$_{12}$H$_{12}$ and KNO$_{3}$), thus broadening significantly the spectrum of caloric materials with commercial potential for solid-state cooling applications. We believe that our simulation study will stimulate experimental research on this new family of barocaloric materials, namely, alkali-metal complex hydrides, which are already known from other technological disciplines (e.g., hydrogen storage and electrochemical devices) and are routinely synthesized in the laboratory.

## Methods

### Classical molecular dynamics simulations

Molecular dynamics (MD) (*N*, *P*, *T*) simulations^60^ were performed with the LAMMPS code^61^. The pressure and temperature in the system were kept fluctuating around a set-point value by using thermostatting and barostatting techniques in which some dynamic variables are coupled to the particle velocities and simulation box dimensions. The interactions between atoms were modeled with the harmonic Coulomb–Buckingham interatomic potential reported in work^38^, the details of which are provided in the Supplementary Methods. The employed interatomic potential reproduces satisfactorily structural properties and lithium diffusion coefficients of bulk LBH as obtained with first-principles methods and experiments (Ref.^38^ and Supplementary Discussion). A particle–particle particle-mesh *k*-space solver was used to compute long-range van der Waals and Coulomb interactions beyond a cut-off distance of 12 Å  at each time step. We employed simulation boxes containing 6656 atoms and applied periodic boundary conditions along the three Cartesian directions. Newton’s equations of motion were integrated by using the customary Verlet’s algorithm with a time-step length of 0.5 fs. In most cases, the simulations were initialized from the ordered $\alpha$ phase. The standard duration of a MD (*N*, *P*, *T*) run was of 2 ns and statistical time averages typically were calculated over the last 1 ns interval. Numerical convergence tests were carried out that demonstrate the adequacy of these technical parameters and statistical procedure (Supplementary Fig. 7).

### Density functional theory calculations

First-principles calculations based on density functional theory (DFT)^42^ were performed to analyse the structural and ionic transport properties of Li$_{2}$B$_{12}$H$_{12}$. We performed these calculations with the VASP software^62^ by following the generalized gradient approximation to the exchange-correlation energy due to Perdew et al.^63^. The projector augmented-wave method was used to represent the ionic cores^64^, and the electronic states 1*s*-2*s* Li, 1*s*-2*s*-2*p* B and 1*s* H were considered as valence. Wave functions were represented in a plane-wave basis set truncated at 650 eV. By using these parameters and dense $\mathbf{k}$-point grids for Brillouin zone integration, the resulting energies were converged to within 1 meV per formula unit. In the geometry relaxations, a tolerance of 0.01 eV Å$^{-1}$ was imposed on the atomic forces.

Ab initio molecular dynamics (AIMD) simulations based on DFT were carried out to reassess the reliability of the interatomic potential model employed in the classical molecular dynamics simulations^38^ (Supplementary Fig. 8 and Supplementary Discussion). The AIMD simulations were performed in the canonical (*N*, *V*, *T*) ensemble, considering constant number of particles, volume and temperature, and were initialized from the ordered $\alpha$ phase. The constrained volumes were equal to the equilibrium volumes determined at zero temperature, thus we neglected possible thermal expansion effects. Nevertheless, in view of previous first-principles work^43^, it is reasonable to expect that thermal expansion effects do not affect significantly the estimation of lithium diffusion coefficients at the considered temperatures. The temperature in the AIMD simulations was kept fluctuating around a set-point value by using Nose–Hoover thermostats. A large simulation box containing 832 atoms was employed in all the simulations, and periodic boundary conditions were applied along the three Cartesian directions. Newton’s equations of motion were integrated by using the customary Verlet’s algorithm and a time-step length of $\delta t = 10^{-3}$ ps. $\Gamma$-point sampling for integration within the first Brillouin zone was employed in all the AIMD simulations. The AIMD simulations comprised long simulation times of $\sim 100$ ps.

### Estimation of key quantities

The mean square displacement of lithium ions was estimated with the formula^43^:

1 $$\begin{aligned} \text{MSD}_\text{Li}(\tau )= & {} \frac{1}{N_\text{ion} \left( N_\text{step} - n_{\tau } \right) } \nonumber \\&\times \sum _{i=1}^{N_\text{ion}} \sum _{j=1}^{N_\text{step} - n_{\tau }} | \mathbf{r}_{i} \left( t_{j} + \tau \right) - \mathbf{r}_{i} \left( t_{j}\right) |^{2}~, \end{aligned}$$

where $\mathbf{r}_{i}(t_{j})$ is the position of the migrating ion *i* at time $t_{j}$ ($= j \cdot \delta t$), $\tau$ represents a lag time, $n_{\tau } = \tau / \delta t$, $N_\text{ion}$ is the total number of mobile ions, and $N_\text{step}$ the total number of time steps. The maximum $n_{\tau }$ was chosen equal to $N_\text{step}/2$, hence we could accumulate enough statistics to reduce significantly the fluctuations in $\text{MSD}_\text{Li}(\tau )$ at large $\tau$’s. The diffusion coefficient of lithium ions then was obtained with the Einstein relation:

2 $$\begin{aligned} D_\text{Li} = \lim _{\tau \rightarrow \infty } \frac{\text{MSD}_{Li}(\tau )}{6\tau }~, \end{aligned}$$

by performing linear fits to the averaged $\text{MSD}_{Li}$ values calculated at long $\tau$.

The angular autocorrelation function of the closoborane (BH)$_{12}^{2-}$ icosahedra was estimated according to the expression^38^:

3 $$\begin{aligned} \phi _{\text{B}_{12}\text{H}_{12}} (\tau ) = \langle \hat{\mathbf{r}} (t) \cdot \hat{\mathbf{r}} (t + \tau ) \rangle ~, \end{aligned}$$

where $\hat{\mathbf{r}}$ is a unitary vector connecting the center of mass of each closoborane unit with one of its edges and $\langle \cdots \rangle$ denotes thermal average considering all the closoborane icosahedra. This autocorrelation function typically decays as $\propto \exp {[-\lambda _{\text{B}_{12}\text{H}_{12}} \cdot \tau ]}$, where the parameter $\lambda _{\text{B}_{12}\text{H}_{12}}$ represents a characteristic reorientational frequency. When the (BH)$_{12}^{2-}$ reorientational motion is significant, that is, $\lambda _{\text{B}_{12}\text{H}_{12}}$ is large, the $\phi _{\text{B}_{12}\text{H}_{12}}$ function decreases rapidly to zero with time. In this case, $\tau$ was chosen equal to 100 ps although statistics were gathered through the whole duration of the MD simulations.

In view of the reported first-order nature of the $\alpha \leftrightarrow \beta$ transformation^32,58^, isothermal entropy changes associated with barocaloric effects were estimated with the Clausius–Clapeyron relation^2,3^:

4 $$\begin{aligned} \Delta S (P,T) = \frac{dP}{dT} (P,T) \cdot \Delta V (P,T)~, \end{aligned}$$

where *dP*/*dT* represents the slope of the coexistence line separating the stability regions of the $\alpha$ and $\beta$ phases, and $\Delta V$ the volume change associated with the phase transition. The dependence of the involved physical quantities on both pressure and temperature are explicitly noted as “(*P*, *T*)”.

Likewise, the accompanying adiabatic temperature shifts were calculated as:

5 $$\begin{aligned} \Delta T (P,T) = - \frac{T}{C_{P}(T)} \cdot \Delta S (P,T)~, \end{aligned}$$

where $C_{P}(T) = \left( \frac{dH}{dT} \right) _{P}$ is the heat capacity of the crystal calculated at constant pressure and temperature and *H* represents the enthalpy of the system. The heat capacity was computed by numerically estimating the temperature derivative of $\langle H \rangle$ at fixed (*P*, *T*) conditions, where $\langle \cdots \rangle$ indicates thermal average. The heat capacity entering Eq. (5) was corrected for latent heat contributions^17,65^ thus for the estimation of $\Delta T$ we adopted $C_{P}$ values obtained at temperatures close to the corresponding transition points (Supplementary Fig. 2; recall that strictly speaking the heat capacity of a first-order transformation diverges at the transition point^65^). Specifically, we found that the corrected $C_{P}$ was quite insensitive to compression and equal to 4100 J K$^{-1}$ kg$^{-1}$ within the analysed pressure and temperature intervals (Supplementary Fig. 2). In order to accurately compute the barocaloric shifts $\Delta S (P, T)$ and $\Delta T (P, T)$ as well as the volume, $D_\text{Li}$, $\lambda _{\text{B}_{12}\text{H}_{12}}$ and $C_{P}$ of bulk LBH, our molecular dynamics calculations were performed for dense grids of (*P*, *T*) points spaced by small increments of $\delta P = 0.05$ GPa and $\delta T = 12.5$ K, respectively.

## Supplementary Information

Supplementary Information 1.

## Data Availability

The data that support the findings of this study are available from the corresponding author (C.C.) upon reasonable request.
