# Supplementary material for: Colossal barocaloric effects in the complex hydride Li2B12H12
Source: Sci Rep. 2021 Jun 7;11:11915. doi: 10.1038/s41598-021-91123-4 (PMC8184963; doi:10.1038/s41598-021-91123-4)
Supplement: Supplementary file 1 — Supplementary Information 1. [file 41598_2021_91123_MOESM1_ESM.pdf]

SUPPLEMENTARY FIGURES

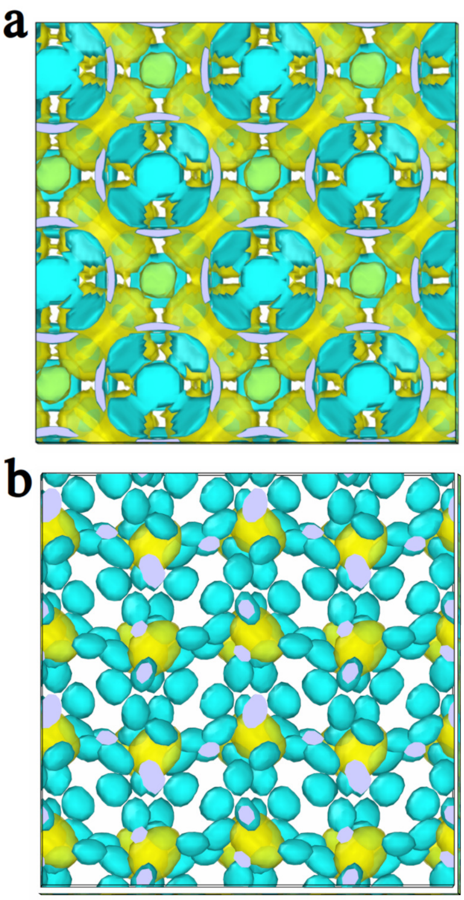

Supplementary Figure 1: Isosurface density plot for Li and H ions with an isovalue of 9×10^{-6} $\AA^{-3}$ (yellow) and 1×10^{-4} $\AA^{-3}$ (cyan), respectively. Purple contours represent the edge of the simulation $2 \times 2 \times 2$ supercell. Results are shown for conditions **a** $P = 0.0$ GPa and $T = 500$ K (β phase) and **b** $P = 0.0$ GPa and $T = 450$ K (α phase). High-density (low-density) Li areas are identified with tetrahedral (octahedral) sites located between $B_{12}H_{12}$ anions, thus Li cations tend to hop from tetragonal to nearest tetragonal sites through closely available disordered sites [1]. These results have been obtained from (N,P,T) molecular dynamics simulations performed with the interatomic potential reported in work [1] (Supplementary Methods).

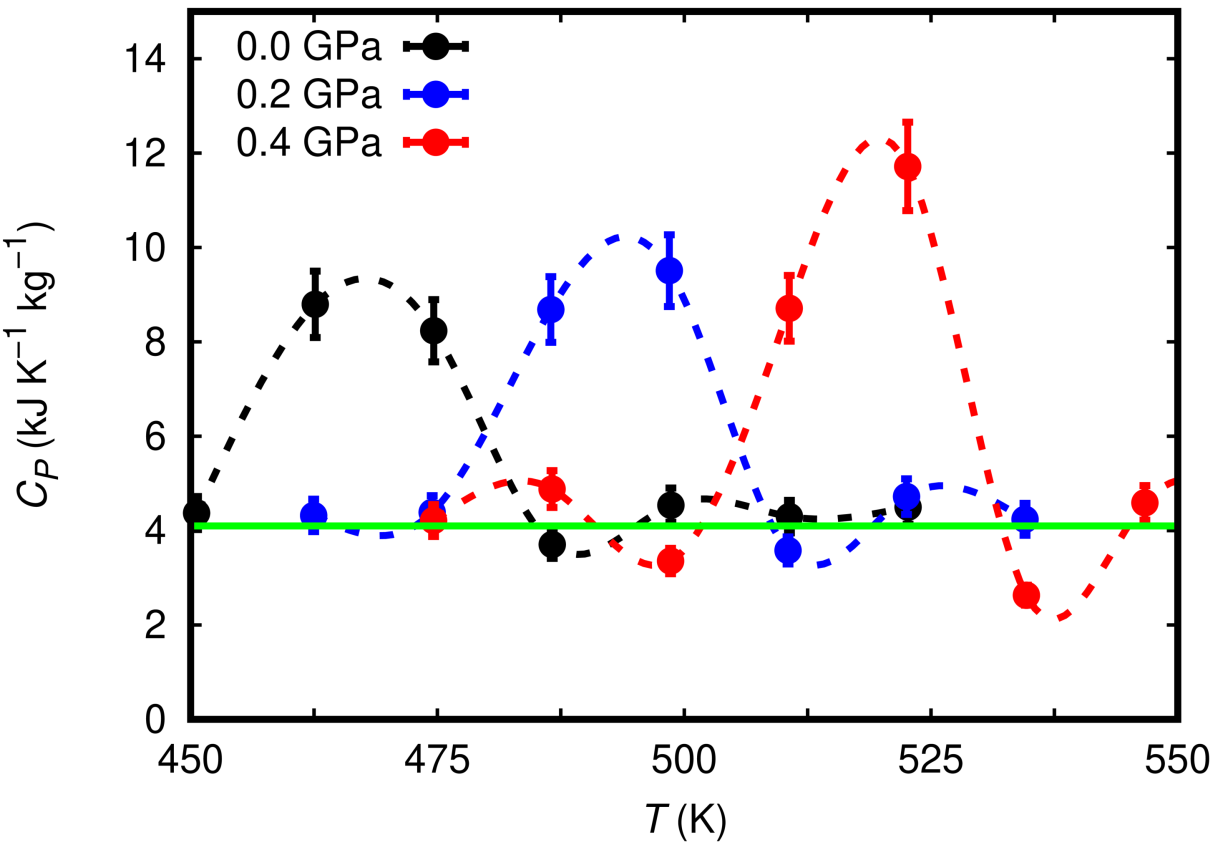

Supplementary Figure 2: Heat capacity of bulk $Li_2B_{12}H_{12}$ expressed as a function of pressure and temperature. The dashed lines are guides to the eye. The C_P values were obtained directly from our (N,P,T) molecular dynamics simulations performed at temperature intervals of 12.5 K and fixed pressure, by numerically estimating the enthalpy of the system and subsequently taking its temperature derivative. The green solid line represents the average C_P value of 4100 JK^{-1}kg^{-1} that is obtained after correcting for the latent heat of the $\alpha \leftrightarrow \beta$ phase transition (i.e., by neglecting the large heat capacity peaks appearing in the plots at the transition points, as it is usually done in calorimetry-based barocaloric experiments [2]). The results have been obtained with the interatomic potential reported in work [1] (Supplementary Methods).

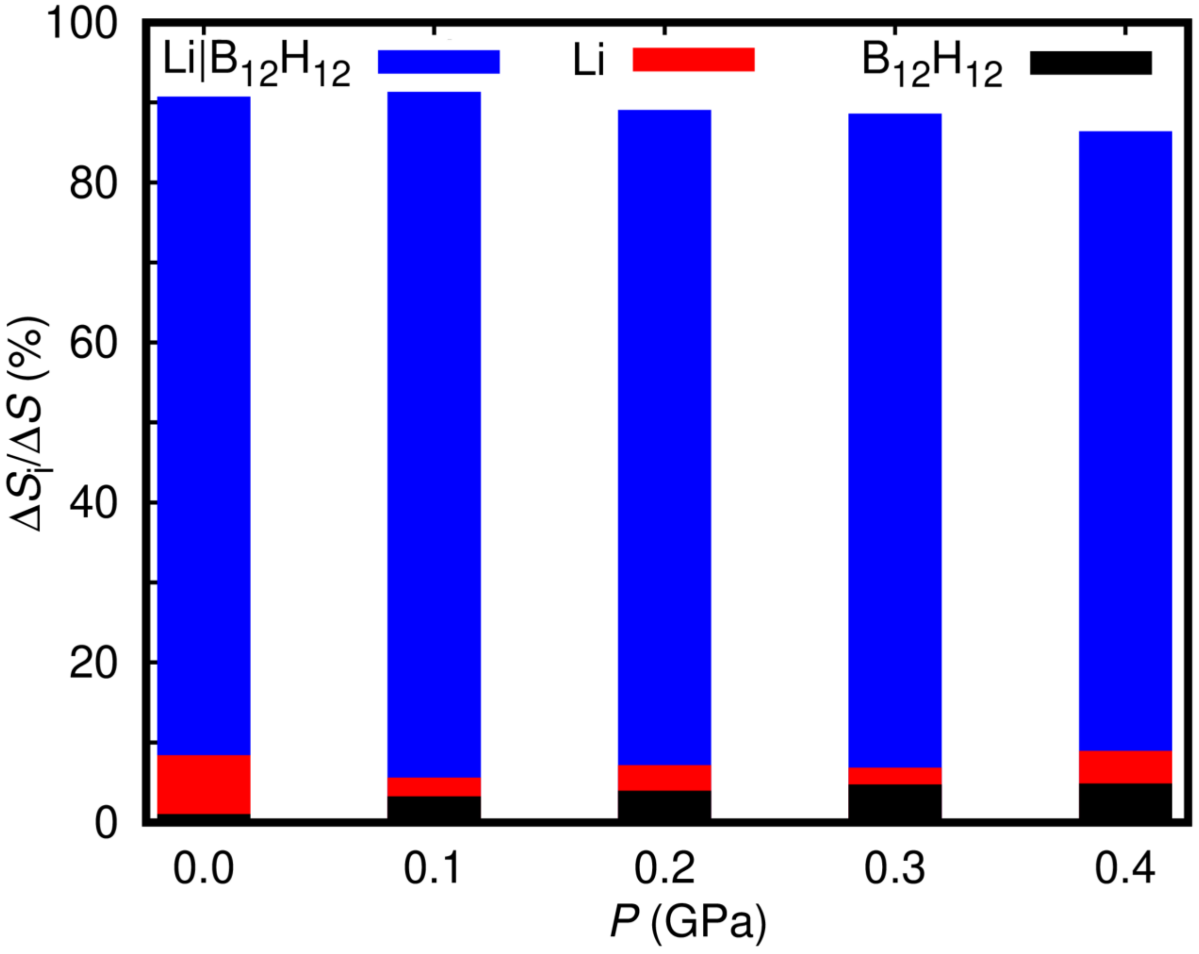

Supplementary Figure 3: Partial contribution to the $\alpha \leftrightarrow \beta$ phase transition entropy expressed as a function of pressure. Red and black rectangles correspond to the partial entropy contribution stemming from "uncorrelated" Li^+ and $(BH)_{12}^{-2}$ ions, respectively (see main text). Blue rectangles correspond to the partial entropy contribution stemming from the interplay between Li^+ and $(BH)_{12}^{-2}$ ions. Li^+ and $(BH)_{12}^{-2}$ ionic correlations (case "$Li|B_{12}H_{12}$") are shown to play a major role on the $\alpha \leftrightarrow \beta$ phase transition. These results have been obtained from (N,P,T) molecular dynamics simulations performed with the interatomic potential reported in work [1] (Supplementary Methods).

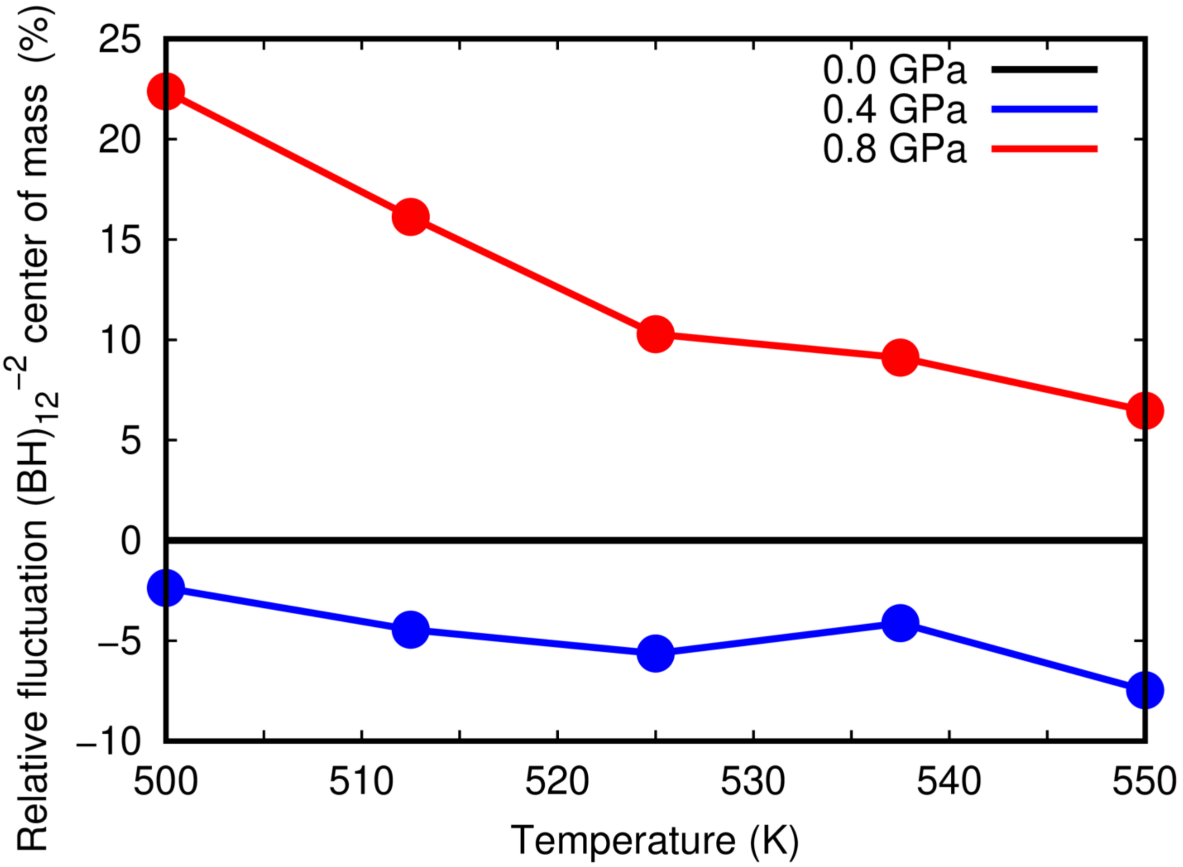

Supplementary Figure 4: Average spatial fluctuation of $(BH)_{12}^{-2}$ anions expressed as a function of pressure and temperature relative to the $P = 0$ GPa case. The average spatial fluctuation of the molecular $(BH)_{12}$ anions is calculated as the standard deviation of the position of their centers of mass (CMs). At $P = 0.4$ GPa, the fluctuation of the $(BH)_{12}^{-2}$ CMs is found to be about $1 - 10\%$ smaller than at zero pressure (depending on temperature). At $P = 0.8$ GPa, the fluctuation of the $(BH)_{12}^{-2}$ CMs is found to be about $5 - 25\%$ larger than at zero pressure (depending on temperature). These results have been obtained from (N,P,T) molecular dynamics simulations performed with the interatomic potential reported in work [1] (Supplementary Methods).

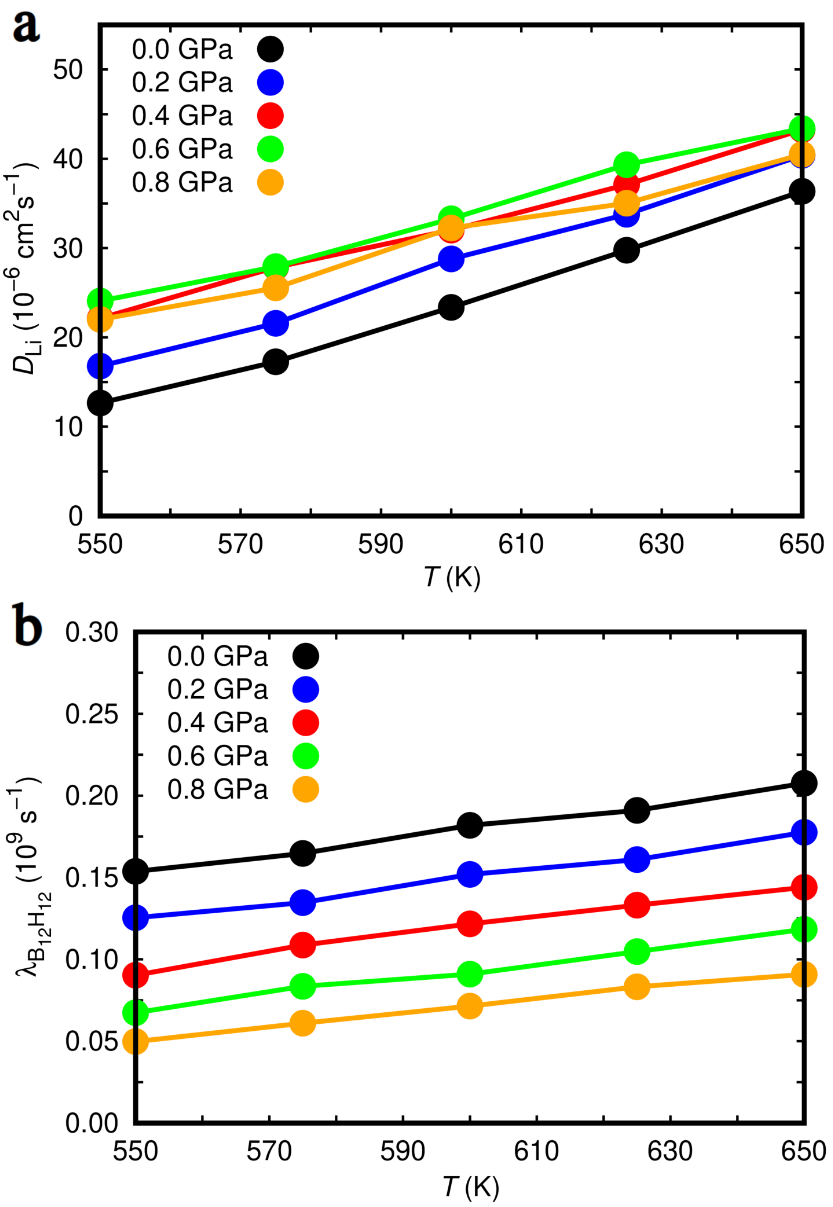

Supplementary Figure 5: Influence of pressure and temperature on bulk $Li_2B_{12}H_{12}$. **a** Lithium ion diffusion coefficient, D_{Li}. **b** $B_{12}H_{12}$ icosahedra reorientational rate, $\lambda_{B_{12}H_{12}}$. These results have been obtained from (N,P,T) molecular dynamics simulations performed with the interatomic potential reported in work [1] (Supplementary Methods).

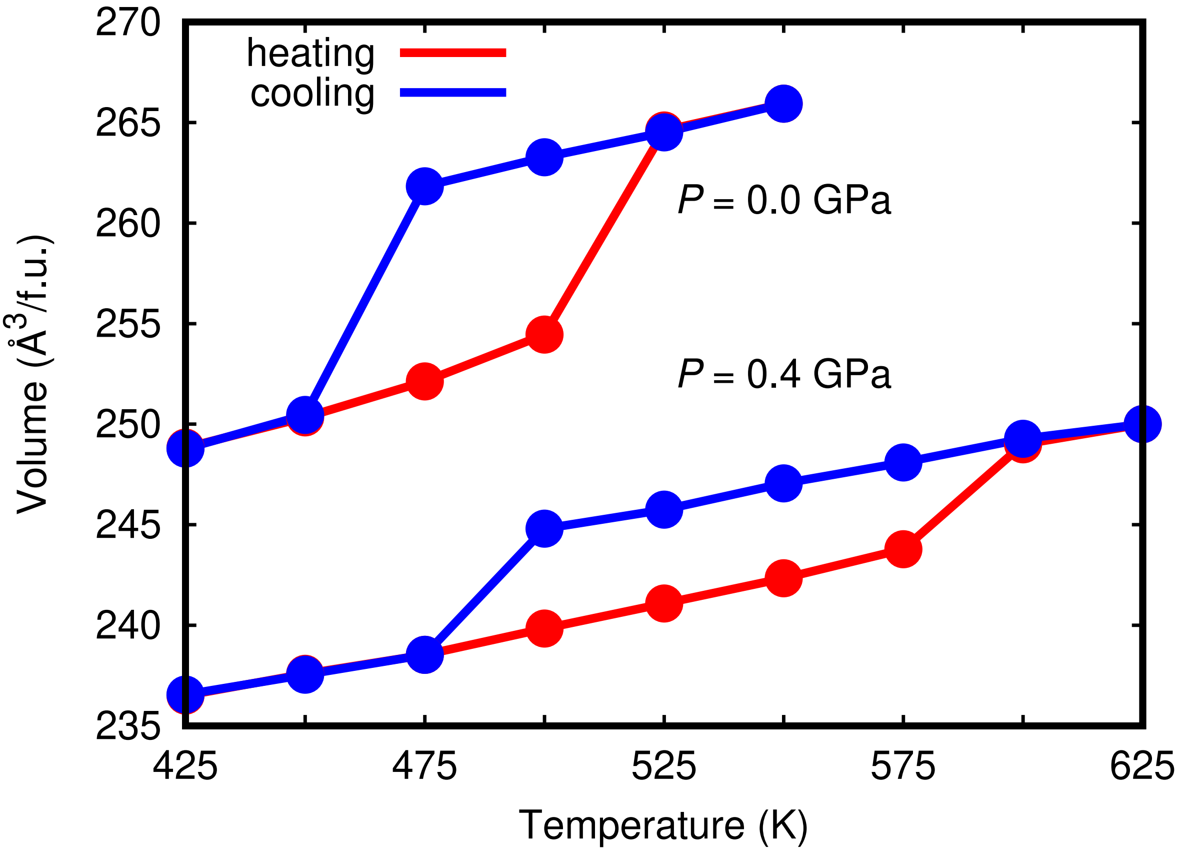

Supplementary Figure 6: Hysteresis of the order-disorder $\alpha \leftrightarrow \beta$ phase transition occurring in bulk $Li_2B_{12}H_{12}$ as estimated from (N,P,T) molecular dynamics simulations performed with the interatomic potential reported in work [1] (Supplementary Methods). The order-disorder $\alpha \leftrightarrow \beta$ phase transition is driven by temperature (i.e., heating-cooling cycles) while pressure is kept fixed. Estimated volumes (solid dots) correspond to average values obtained over short time intervals of ~ 1 ps separated by ~ 100 ps. The system is not legitimately equilibrated since the temperature changes at a constant rate of 100 K/ns; thus, these results cannot be directly compared to those shown in Figs.2 and 4 of the main text since the latter were obtained under equilibrium conditions.

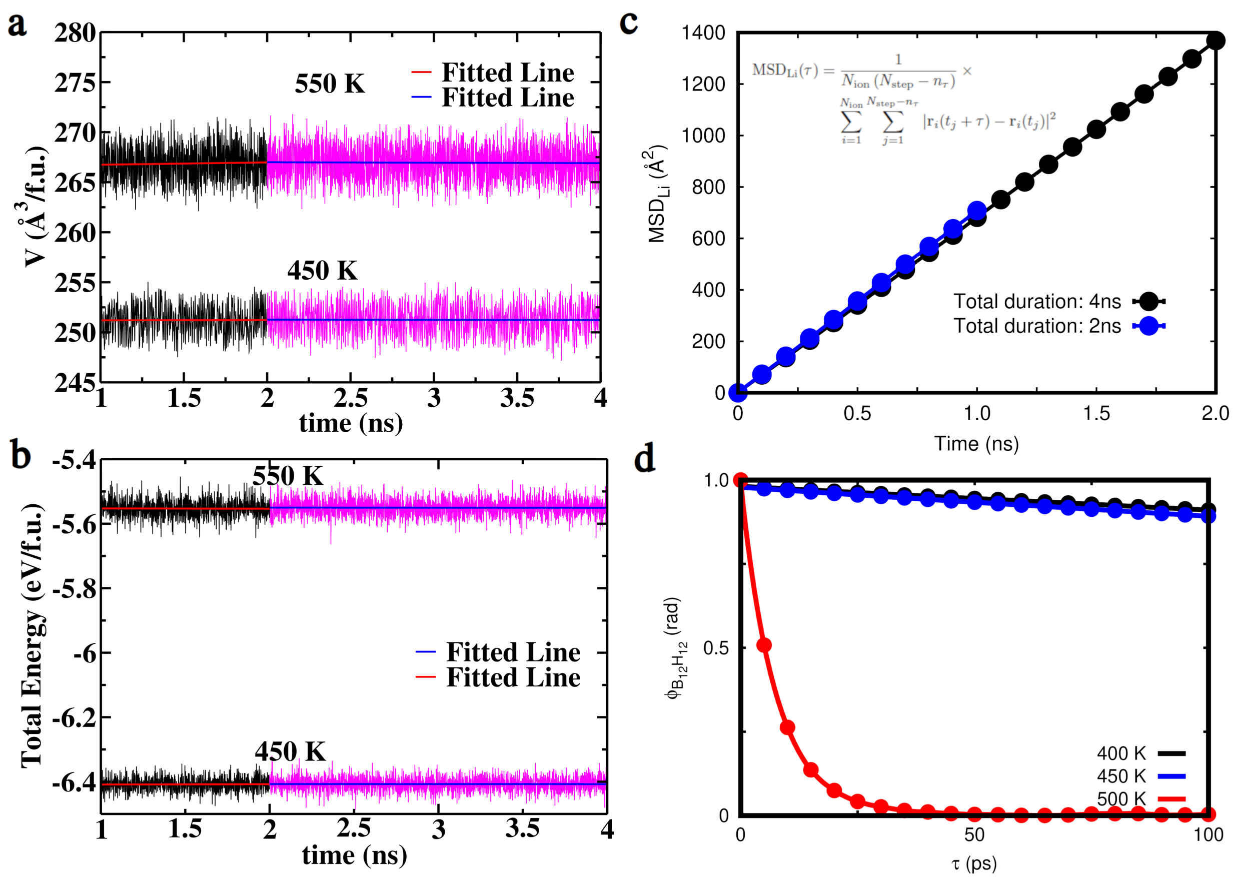

Supplementary Figure 7: Convergence tests for our classical molecular dynamics (MD) simulations. The structural, energy and dynamical properties of the system are shown to be properly converged for a typical MD run of 2 ns duration. Numerical fits are shown to reproduce very accurately the sets of data that have been explicitly calculated (solid circles). These results have been obtained from (N,P,T) MD simulations performed with the interatomic potential reported in work [1] (Supplementary Methods).

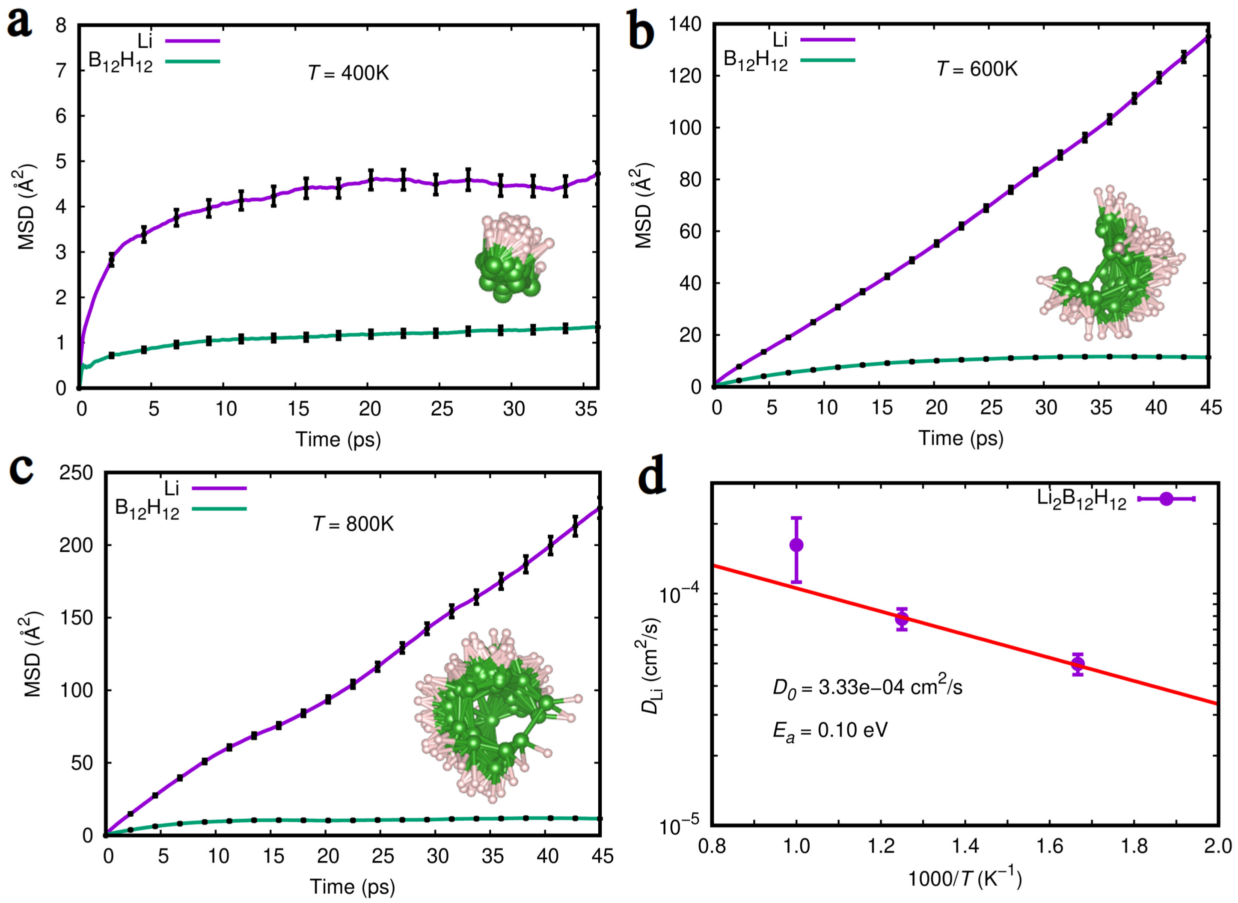

Supplementary Figure 8: Mean-squared displacement, MSD, estimated for bulk $Li_2B_{12}H_{12}$ from *ab initio* molecular dynamics simulations (AIMD) [3] based on density functional theory [4] at **a** $T = 400$ K, **b** 600 K and **c** 800 K (Supplementary Discussion). The orientation of an arbitrary B–H bond taken from an arbitrary $B_{12}H_{12}$ icosahedron is also monitored as a function of temperature. B and H atoms are represented by green and pink spheres, respectively. The lithium ion diffusion coefficients, D_{Li}, calculated from the corresponding MSD curves are shown in **d** (Methods).

SUPPLEMENTARY METHODS

For our molecular dynamics (MD) simulations of bulk $Li_2B_{12}H_{12}$ (LBH) we adopted the force field reported in work [1]. This force field has been already shown to provide a satisfactory description of the vibrational, structural and ionic diffusion properties of bulk LBH [1], and we have further tested it here against additional *ab initio* molecular dynamics simulations (Supplementary Discussion). The adopted LBH force field consists of a Coulomb-Buckingham and harmonic type interatomic potential of the form:

$$U_{\rm LBH}(\mathbf{r}_1, \mathbf{r}_2, \cdots, \mathbf{r}_N) = \sum_{i>j=1}^{N} U_{\rm non-bonded}(r_{ij}) + \sum_{i>j=1}^{N} U_{\rm bond}(d_{ij}) + \sum_{i>j>k=1}^{N} U_{\rm angle}(\theta_{ijk}) \ , \quad (1)$$

where N is the total number of ions and each of the energy terms above is expressed as:

$$U_{\rm non-bonded}(r_{ij}) = \frac{q_i q_j}{r_{ij}} + A_{ij} e^{-r_{ij}/\rho_{ij}} - \frac{C_{ij}}{r_{ij}^6}, \quad (2)$$

$$U_{\rm bond}(d_{ij}) = \frac{1}{2} k_{ij} \left(d_{ij} - d_{ij}^0 \right)^2, \quad (3)$$

$$U_{\rm angle}(\theta_{ijk}) = \frac{1}{2} k_{ijk} \left(\theta_{ijk} - \theta_{ijk}^0 \right)^2 \ . \quad (4)$$

In the equations above, the distance $r_{ij} \equiv |\mathbf{r}_i - \mathbf{r}_j|$ runs over all possible couple of ions, the distance $d_{ij} \equiv |\mathbf{r}_i - \mathbf{r}_j|$ runs over all possible couple of nearest neighbours B–B and B–H ions, and the angle θ_{ijk} runs over all possible trio of nearest neighbours B–H–B ions. The numerical value of the interatomic potential parameters are the following (if the parameter is not listed, its value is zero) [1]:

$$\begin{aligned}
q_{\rm Li} &= 0.85, q_{\rm H} = -0.1417 \\
A_{\rm Li-B} &= 7793.7, A_{\rm Li-H} = 7375.3, A_{\rm H-H} = 209905.0 \\
\rho_{\rm Li-Li} &= 0.4000, \rho_{\rm Li-B} = 0.2853, \rho_{\rm Li-H} = 0.3023, \rho_{\rm B-H} = 0.2853, \rho_{\rm H-H} = 0.2085, \\
C_{\rm B-B} &= 405.848, C_{\rm H-H} = 418.4, \\
k_{\rm B-B} &= 125.0, d^0_{\rm B-B} = 1.79, k_{\rm B-H} = 962.3, d^0_{\rm B-H} = 1.20, \\
k_{\rm B-H-B} &= 83.7 \text{ and } \theta^0_{\rm B-H-B} = 2.0944, \quad (5)
\end{aligned}$$

where the corresponding units are, $[q] = e$, $[A] =$ kJ mol^{-1}, $[\rho] =$ Å, $[C] =$ Å^6 kJ mol^{-1}, $[k_{ij}] =$ Å^{-2} kJ mol^{-1}, $[d^0_{ij}] =$ Å, $[k_{ijk}] =$ rad^{-2} kJ mol^{-1} and $[\theta^0_{ijk}] =$ rad.

SUPPLEMENTARY DISCUSSION

Ab initio molecular dynamics (AIMD) simulations based on DFT [4] were performed to further assess the reliability of the interatomic potential model employed in the classical molecular dynamics simulations. The AIMD simulations were performed in the canonical (N, V, T) ensemble considering constant number of particles, volume and temperature. The constrained volumes were equal to the equilibrium volumes determined at zero temperature, thus we neglected possible thermal expansion effects. Nevertheless, in view of previous first-principles work [3], it is reasonable to expect that thermal expansion effects do not affect significantly the estimation of lithium diffusion coefficients at the considered temperatures. The temperature in the AIMD simulations was kept fluctuating around a set-point value by using Nose-Hoover thermostats. A large simulation box containing 832 atoms was employed in all the simulations, and periodic boundary conditions were applied along the three Cartesian directions. Newton's equations of motion were integrated by using the customary Verlet's algorithm and a time-step length of $\delta t = 10^{-3}$ ps. Γ-point sampling for integration within the first Brillouin zone was employed in all the AIMD simulations. The AIMD simulations comprised long simulation times of ~ 100 ps. We performed these calculations with the VASP software [5] by following the generalized gradient approximation to the exchange-correlation energy due to Perdew *et al.* [6]. The projector augmented-wave method was used to represent the ionic cores [7], and the electronic states $1s$-$2s$ Li, $1s$-$2s$-$2p$ B and $1s$ H were considered as valence. Wave functions were represented in a plane-wave basis set truncated at 650 eV. The resuts of our AIMD simulations for bulk LBH are summarized in Supplementary Fig.8.

In consistent agreement with the force field results, we found that the disordered β phase is stabilized over the ordered α phase at temperatures higher than 400 K. In particular, the diffusion coefficient of the Li^+ ions, $D_{\rm Li}$, is practically zero at $T = 400$ K whereas at temperatures of 600, 800 and 1000 K amounts to $4.97 \cdot 10^{-5}$, $7.79 \cdot 10^{-5}$ and $1.62 \cdot 10^{-4}$ cm^{2}s^{-1}, respectively (Supplementary Fig.8a-d). We also confirmed the activation of $(BH)_{12}^{-2}$ reorientational motion at temperatures above 400 K by monitoring the trajectory of an arbitrary B–H bond taken from an arbitrary icosahedron anion (Supplementary Fig.8a-c). In this case, we did not attempt to estimate the $(BH)_{12}^{-2}$ icosahedra reorientational frequency due to the prohibitively long AIMD simulation times that are necessary for such

type of calculation [1].

By assuming that the T-dependence of the Li^+ diffusion coefficient follows the Arrhenius formula [3]:

$$D_{\text{Li}}(T) = D_0 \cdot \exp\left[-\frac{E_a}{k_B T}\right] , \tag{6}$$

where k_B is the Boltzmann constant, we obtained a fitted pre-exponential factor of $D_0^{\text{AIMD}} = 3.33 \cdot 10^{-4}$ cm^{2}s^{-1} and an activation energy for ionic migration of $E_a^{\text{AIMD}} = 0.10$ eV. The D_{Li} values calculated with AIMD simulations compare reasonably well with the zero-pressure results obtained with classical molecular dynamics (MD) and the force field described in the Supplementary Methods. In particular, the Li^+ diffusion coefficient estimated with MD simulations at $T = 500$, 600 and 700 K are, respectively, $4.88 \cdot 10^{-6}$, $2.33 \cdot 10^{-5}$ and $4.89 \cdot 10^{-5}$ cm^{2}s^{-1}. By assuming an Arrhenius-like T-dependence for D_{Li} also in this case, we obtained a fitted pre-exponential factor of $D_0^{\text{MD}} = 9.54 \cdot 10^{-4}$ cm^{2}s^{-1} and an activation energy for ionic migration of $E_a^{\text{MD}} = 0.22$ eV.

Overall, the phase transition and Li^+ diffusion coefficient results obtained with MD techniques are consistent, although not identical, with those found in AIMD simulations (as expected). Thus, the reliability of the employed force field to describe the structural, phase transition and barocaloric properties of bulk $Li_2B_{12}H_{12}$ can be regarded as reasonably high (as previously concluded in work [1] and further discussed in the main text of the present study).

SUPPLEMENTARY REFERENCES

[1] Sau, K., Ikeshoji, T., Kim, S., Takagi, S., Akgi, K. & Orimo, S.-i. Reorientational motion and Li^+ ion trasnport in $Li_2B_{12}H_{12}$ system: Molecular dynamics study. *Phys. Rev. Mater.* **3**, 075402 (2019).

[2] Aznar, A., Lloveras, P., Romanini, M., Barrio, M., Tamarit, J. Ll., Cazorla, C., Errandonea, D., Mathur, N. D., Planes, A., Moya, X. & Mañosa, Ll. Giant barocaloric effects over a wide temperature range in superionic conductor AgI. *Nat. Commun.* **8**, 1851 (2017).

[3] Sagotra, A. K., Chu, D. & Cazorla, C. Influence of lattice dynamics on lithium-ion conductivity: A first-principles study. *Phys. Rev. Mater.* **3**, 035405 (2019).

[4] Cazorla, C. & Boronat, J. Simulation and understanding of atomic and molecular quantum crystals. *Rev. Mod. Phys.* **89**, 035003 (2017).

[5] Kresse, G. & Furthmüller, J. Efficient iterative schemes for ab initio total-energy calculations using a plane-wave basis set. *Phys. Rev. B* **54**, 11169 (1996).

[6] Perdew, J. P., Burke, K. & Ernzerhof, M. Generalized gradient approximation made simple. *Phys. Rev. Lett.* **77**, 3865 (1996).

[7] Blöchl, P. E. Projector augmented-wave method. *Phys. Rev. B* **50**, 17953 (1994).
